# Accuracy of ophthalmic referral diagnoses by non-ophthalmologists in acute eye care: protocol for a systematic review and meta-analysis

**DOI:** 10.1136/bmjopen-2026-121497

**Published:** 2026-06-12

**Authors:** Luis Cunha Gil, Anaya Powis, Helen Wilson, Reshma Thampy, Obaid Kousha, Felipe Dhawahir-Scala

**Affiliations:** 1 University of Manchester - The Victoria University of Manchester Campus, Manchester, Greater Manchester, UK; 2 Manchester Royal Eye Hospital, Manchester, England, UK; 3 Infection and Global Health Division, School of Medical and Biological Sciences, University of St Andrews, St Andrews, Scotland, UK

**Keywords:** OPHTHALMOLOGY, ACCIDENT & EMERGENCY MEDICINE, Systematic Review, Meta-Analysis

## Abstract

**Abstract:**

**Introduction:**

Ophthalmic complaints account for a substantial proportion of presentations to emergency and acute eye care services, yet initial assessment or referral is frequently performed by non-ophthalmologist healthcare professionals. Previous single-centre studies suggest that one-third of referrals are incorrectly diagnosed, potentially delaying appropriate management of vision-threatening conditions. However, the overall magnitude of diagnostic error and patterns of misdiagnosis across healthcare settings remain unclear. This study aims to systematically review and synthesise the evidence on the diagnostic concordance of ophthalmic referral diagnoses made by non-ophthalmologists in acute eye care.

**Methods and analysis:**

A systematic review and meta-analysis will be conducted following PRISMA (Preferred Reporting Items for Systematic Reviews and Meta-Analysis Protocols) guidance and registered with PROSPERO. MEDLINE (Ovid), Embase (Ovid) and the Cochrane CENTRAL database will be searched from inception to April 2025. Studies evaluating the diagnostic accuracy of referrals made by non-ophthalmologist healthcare professionals in emergency or acute eye care settings will be included. Two reviewers will independently screen studies, extract data and assess risk of bias using the QUADAS-2 (Quality Assessment of Diagnostic Accuracy Studies-2) framework adapted for referral-diagnosis studies. The primary outcome will be diagnostic concordance between referral and final ophthalmologist diagnosis. Where appropriate, pooled concordance proportions will be synthesised using a random-effects meta-analysis. Condition-specific 2×2 diagnostic accuracy analyses will only be undertaken where valid binary target conditions and sufficient denominators are reported. Heterogeneity will be assessed using Cochran’s Q test and the I² statistic with subgroup analyses exploring differences by referring clinician type and anatomical location of ophthalmic pathology.

**Ethics and dissemination:**

Ethical approval is not required for this study as it will synthesise data from previously published studies; findings will be disseminated through publication in a peer-reviewed journal and presentation at relevant academic conferences.

**PROSPERO registration number:**

CRD420261352717.

**STRENGTHS AND LIMITATIONS OF THIS STUDY**

- The review will follow the Preferred Reporting Items for Systematic Reviews and Meta-Analysis Protocols (PRISMA-P) guidance and a pre-registered protocol on (PROSPERO).
- Duplicate independent screening, data extraction and risk-of-bias assessment will reduce selection bias and extraction error, with disagreements resolved by a third reviewer.
- A structured analytical hierarchy will be used, with pooled diagnostic concordance proportions as the primary outcome and condition-specific 2×2 diagnostic accuracy analysis as a secondary measure where methodologically valid.
- Considerable heterogeneity in diagnostic classification systems, referral pathways and study design across centres may limit the comparability of studies and restrict the quantitative synthesis.
- Restriction to English language full-text studies may introduce language bias and limit representation of non-English-speaking healthcare systems.

## Introduction

 Accident and emergency departments face the major challenge of undifferentiated presentations, approximately 1–6% of which are accounted for by ophthalmic conditions as per current estimates.^1^ Despite initiatives such as Minor Eye Care Schemes and the development of dedicated emergency eye departments, healthcare systems in the UK and globally continue to face increasing demand for both emergency and routine ophthalmic services, contributing to delays in care and preventable visual morbidity.^2 3^ In England, ophthalmology represented 8.5% of all NHS (National Health Service) referral-to-treatment waiting list activity in 2023/2024, with more than 586 000 patients waiting for ophthalmology appointments in March 2025.^4^ With a widening disparity between number of ophthalmologists and the prevalence of eye disease, current advice from the Royal College of Ophthalmologists highlights the expanding role of community-based healthcare professionals in managing acute eye conditions and facilitating appropriate referral pathways.^1^

In many healthcare systems, patients presenting with acute eye symptoms are initially assessed by non-ophthalmologist clinicians, such as general practitioners, emergency physicians, physician associates and nurses, before referral for specialist ophthalmic evaluation. However, previous studies across multiple centres consistently report referral misdiagnosis rates of around one-third when compared with the diagnosis established by an ophthalmologist, treated as the reference standard.^5^,^9^ These findings are consistent with studies demonstrating that emergency physicians and general practitioners frequently report limited confidence in assessing and managing acute eye presentations.^10 11^ Importantly, diagnostic discordance has been observed even in referrals made between ophthalmologists, with approximately one in five inter-ophthalmology referrals differing from the final diagnosis.^6^

Up to 13% of eye presentations to acute care settings represent true ophthalmic emergencies which progress rapidly to threaten vision, thus requiring accurate identification and timely intervention to prevent irreversible vision loss.^12^ True ocular emergencies include conditions such as acute angle-closure glaucoma, retinal detachment, microbial keratitis, endophthalmitis and retinal vascular occlusive disease.^13 14^ Delays in specialist assessment and treatment are associated with poorer visual outcomes across these conditions, driven partially by diagnostic uncertainty or inappropriate referral, particularly in non-specialist settings.^15^ Appropriate identification of ocular findings is also important in the context of other specialities, including rheumatology and neurology, in which ocular manifestations may represent particular underlying disease processes.^16 17^ Consequently, accurate diagnosis at the point of referral is critical, as it may influence urgency of triage, timeliness of specialist assessment and initiation of appropriate management.^15^

Existing evidence on ophthalmic referral diagnostic accuracy is largely derived from single-centre studies, with variation in referrer type, clinical setting, diagnostic classification and case mix. As a result, the overall magnitude of diagnostic discordance and the patterns of misdiagnosis across acute eye care pathways remain unclear. A systematic review and meta-analysis is therefore warranted to synthesise the available evidence, estimate referral diagnostic concordance where appropriate and identify areas where training, triage and referral pathways may be improved.

The objective of this review is to assess the diagnostic concordance of referral diagnoses made by non-ophthalmologists across acute ophthalmic care settings, using the final ophthalmologist diagnosis as the reference standard, with outcomes including referral diagnostic concordance and subgroup analyses by referral setting, clinician type, anatomical patterns of pathology and condition.

## Methods

This systematic review will be conducted and reported following the Preferred Reporting Items for Systematic Reviews and Meta-Analysis Protocols (PRISMA-P) guidelines.^18^ The eligibility criteria and search strategy were informed by the PICO framework (Population, Intervention, Comparison, Outcomes), typically used in treatment-focused systematic reviews.

### Eligibility criteria

#### Types of participants

Studies will be considered for inclusion if they analysed patients initially assessed or referred by non-ophthalmologist healthcare providers (emergency physicians, general practitioners, optometrists, other non-specialists). Optometrist referral data will be extracted and analysed as a distinct referrer subgroup where data permits, given the differences in ophthalmic training and role of optometrists in delivering primary eye care in certain countries. Studies involving mixed referrer groups will be included if data for non-ophthalmologist referrers can be extracted separately or if the non-ophthalmologist group forms the primary group of interest. We will include studies from any emergency or acute eye care setting and categorise the setting receiving the referral as either (1) hospital emergency departments, (2) emergency eye departments/eye casualty services, (3) emergency eye clinics, (4) community urgent eye care pathways or (5) tele-triage services where reported. Studies that focus on patients assessed only by ophthalmologists or routine outpatient ophthalmology care will be excluded.

#### Index diagnosis

The index diagnosis will be the initial/referral diagnosis made by a non-ophthalmologist. Referral diagnoses which are considered anatomically or clinically appropriate, but which lack specific diagnostic terminology, will be recorded as partially concordant. Studies that do not report comparison between referral and final diagnosis will be excluded.

#### Comparison

The reference standard against which we will make a comparison will be the final diagnosis established by the ophthalmologist.

#### Types of outcomes

The primary outcome of our study will be diagnostic concordance between the referral diagnosis made by a non-ophthalmologist and the final diagnosis as confirmed by an ophthalmologist. We will include studies that report raw data or statistics related to diagnostic concordance. Studies using a diagnostic classification system to subdivide accuracy, such as correct, incorrect, non-specific, not yet diagnosed, partially concordant or similar categories, will have definitions extracted as recorded. This diagnostic data will then be grouped as concordant, partially concordant or discordant where possible to facilitate quantitative analysis of outcomes.

Our secondary objectives will include condition-specific sensitivity and specificity where 2×2 data can be validly derived; diagnostic concordance rates by type of referring non-ophthalmologist; ophthalmic conditions most commonly misdiagnosed; and anatomical location of ophthalmic complaints.

#### Types of studies

Only non-randomised study types will be included. We will exclude case reports, case series with less than 10 patients, review articles, conference abstracts without the full data necessary for analysis and studies not written in English. Only English language full-text studies will be included due to the absence of translation resources. This restriction will be acknowledged as a potential source of language bias in the review.

### Information sources

A systematic search will be performed in MEDLINE (Ovid), Embase (Ovid) and CENTRAL Cochrane to search for literature published from database inception until 12 April 2025, which will be updated prior to manuscript submission. The reference lists of all included studies will be manually searched for additional relevant papers.

### Search strategy

A search strategy was developed based on the aforementioned PICO framework, in line with the eligibility criteria. Relevant terms focused on (1) diagnostic accuracy; (2) eye disease; (3) non-ophthalmologist; (4) emergency eye care were developed and searched as controlled vocabulary (Medical Subject Headings (MeSH)) and text words. The study will cover from database inception to 12 April 2025 with limitations set to exclude non-English language papers. The full search strategy is available in the online supplemental material.

### Data records and management

All records identified through database searches will be imported into Endnote software for storage and duplicate removal. Screening of titles, abstracts and full texts will be managed using Rayyan. Extracted data will be recorded in Excel in a structured spreadsheet.

Two independent reviewers (LCG and AP) will screen article titles and abstracts according to the eligibility criteria. The remaining full texts will again be screened against the same inclusion/exclusion criteria. Any conflicts will be resolved by discussion between reviewers with discrepancy of opinion addressed by RT and FD-S. A PRISMA flow chart will be used to depict the details of the selection process.

Two reviewers will independently extract data from eligible studies using a standardised extraction form determined a priori. A random sample of approximately 20% of included papers will be used to ensure consistency and reliability between reviewers, with disagreements resolved through discussion or, where necessary, consultation with a third reviewer.

The following information will be extracted:

1. General (first author, publication date, country of study, digital object identifier (DOI)).
2. Study characteristics (study design, clinical setting, recruitment methods, inclusion and exclusion criteria).
3. Participant characteristics (total number of patients, number of referrals analysed, age (mean or range) and gender where reported).
4. Referring clinician characteristics (type of referring healthcare professional, eg, emergency physician, general practitioner, optometrist, other).
5. Diagnostic assessment (referral diagnosis (index diagnosis), final diagnosis made by ophthalmologist (reference standard), diagnostic classification system used in study, eg, concordant, discordant, partially concordant, non-specific, not yet diagnosed or equivalent categories as defined by the study).
6. Ophthalmic condition characteristics (anatomical location of pathology, eg, retina, cornea, anterior segment, conjunctiva, external eye, neuro-ophthalmic, orbit, other).
7. Primary and secondary outcomes (number of concordant, partially concordant and discordant diagnoses; pooled diagnostic concordance proportion by study; condition-specific sensitivity and specificity where 2×2 data can be validly derived; diagnostic concordance by referring clinician type where available; diagnostic concordance by anatomical location of pathology where available; and most frequently misdiagnosed conditions).
8. Risk of bias information for QUADAS-2 (method of patient selection, blinding between referral diagnosis and ophthalmologist diagnosis, completeness of outcome reporting, time interval between referral and ophthalmologist assessment).

### Risk of bias

Two independent reviewers (LCG and AP) will assess risk of bias using the Quality Assessment of Diagnostic Accuracy Studies-2 (QUADAS-2) tool, with disagreements resolved by discussion or consultation with a third reviewer (RT or FD-S). QUADAS-2 will be tailored to referral-diagnosis studies. In the patient selection domain, low risk will require consecutive or clearly representative acute ophthalmic referrals without inappropriate exclusions; selected samples or exclusion of clinically relevant referral categories will be judged at higher or unclear risk. In the index diagnosis domain, low risk will require the non-ophthalmologist referral diagnosis to be documented before specialist assessment; retrospectively reconstructed diagnoses or diagnoses influenced by ophthalmologist findings will be judged at higher risk. In the reference standard domain, low risk will require a final diagnosis made by an ophthalmologist or supervised ophthalmology trainee following specialist assessment; non-specialist, administrative-code-based, incomplete or poorly described reference diagnoses will be judged at higher or unclear risk. In the flow and timing domain, low risk will require all included patients to receive specialist assessment within an appropriate acute-care timeframe and be included in the analysis; substantial exclusions, missing diagnoses or delays likely to alter diagnosis will be judged at higher or unclear risk. Each domain will be rated as low, high or unclear risk of bias, with applicability concerns assessed for patient selection, index diagnosis and reference standard. Risk-of-bias findings will be tabulated, incorporated into interpretation and explored in sensitivity analyses where sufficient data are available.

### Data synthesis

Quantitative data will be synthesised using meta-analysis where studies are sufficiently homogeneous in terms of population, study design and outcome reporting. The primary quantitative summary measure across all included studies will be the proportion of diagnostic concordance between the referral diagnosis made by a non-ophthalmologist and the final diagnosis confirmed by an ophthalmologist. This measure is calculable regardless of how individual studies classify diagnostic outcomes and does not require definition of a binary target condition. Pooled concordance proportions will be calculated using a random-effects model to account for expected clinical and methodological heterogeneity between studies.

Construction of 2×2 contingency tables consisting of true positives (TP), false positives (FP), true negatives (TN) and false negatives (FN) will be undertaken only as a secondary, condition-specific analysis. In a broad multi-condition referral setting, TN cannot be validly defined across the full case mix because the denominator of patients not referred with a given condition is not consistently reported and varies substantially by setting and case mix. A valid 2×2 classification requires a clearly defined binary target condition, where it is possible to identify patients correctly and incorrectly classified both as having and as not having that specific condition. This form of analysis will therefore only be undertaken where studies report data stratified by clearly defined target conditions with sufficient denominators to permit valid diagnostic classification. Where sufficient comparable studies report condition-specific sensitivity and specificity, bivariate random-effects or hierarchical summary receiver operating characteristic (ROC) methods will be considered in preference to univariate pooling to appropriately account for the correlation between sensitivity and specificity across studies.

Statistical heterogeneity will be assessed using Cochran’s Q test and quantified using the I² statistic, which estimates the proportion of variability across studies attributable to true differences rather than sampling error. Values of approximately 25%, 50% and 75% will be interpreted as representing low, moderate and high heterogeneity, respectively.

Where sufficient data are available, subgroup analyses will explore differences in diagnostic concordance by referring clinician type and anatomical location of the ophthalmic pathology. Sensitivity analyses will be conducted excluding studies judged to be at high risk of bias. If quantitative synthesis is not appropriate due to substantial heterogeneity or insufficient data, findings will be summarised narratively.

### Meta-bias

The primary approach to assessing publication bias and selective outcome reporting will be a qualitative comparison of reported outcomes against study methods and, where available, against prospectively registered protocols, to identify discrepancies suggestive of selective reporting. As a supplementary exploratory analysis, funnel plots and the Egger test will be constructed where sufficient studies (≥10) are available for a given outcome. However, these methods were developed primarily for intervention meta-analyses and have well-recognised limitations in diagnostic accuracy and concordance data: funnel asymmetry may reflect genuine clinical heterogeneity in case mix, setting or referral threshold rather than publication bias; the Egger test assumes a linear relationship between effect size and SE that does not hold for bounded concordance proportions or correlated sensitivity-specificity pairs; and both methods have reduced power and interpretability in the context of high between-study heterogeneity. Results of these supplementary analyses will therefore be interpreted cautiously and considered alongside the qualitative assessment rather than as standalone indicators of publication bias.

### Confidence in cumulative evidence

The certainty of evidence will be assessed using the Grading of Recommendations Assessment, Development and Evaluation (GRADE) approach. Certainty will be assessed separately for each main diagnostic outcome or diagnostic category using the GRADE approach, which rates evidence as high, moderate, low or very low certainty based on five domains including risk of bias, inconsistency, indirectness, imprecision and publication bias.

### Patient and public involvement

Patients or the public were not involved in the design, conduct, reporting or dissemination plans of our research.

## Ethics and dissemination

Ethical approval is not required for this systematic review as it will analyse data from previously published studies and will not involve the collection of primary patient data. The findings will be disseminated through publication in a peer-reviewed journal and presentation at relevant academic conferences.

## Supplementary material

10.1136/bmjopen-2026-121497 online supplemental file 1
