# Supplementary material for: Accuracy of ophthalmic referral diagnoses by non-ophthalmologists in acute eye care: protocol for a systematic review and meta-analysis
Source: BMJ Open. 2026 Jun 12;16(6):e121497. doi: 10.1136/bmjopen-2026-121497 (PMC13289109; doi:10.1136/bmjopen-2026-121497)
Supplement: online supplemental file 1 [file bmjopen-16-6-s001.docx]

**Accuracy of ophthalmic referral diagnoses by non-ophthalmologists in acute eye care: a systematic review and meta-analysis**

Proposed Search Strategy:

The following databases will be searched: MEDLINE, Embase via Ovid, CENTRAL Cochrane; key words will be searched for in the abstracts. The strategy combines four concepts: referral diagnostic accuracy, ophthalmology/eye disease, non-ophthalmologist referrers, and acute/emergency eye care settings. Controlled vocabulary terms and free-text terms were combined to improve sensitivity. The search strategy will be adapted for Embase and CENTRAL using database-specific syntax.

Reference lists and relevant journals will be searched to identify potential eligible studies.

| \| Line \| Search term \| \| --- \| --- \| \| 1 \| exp Diagnostic Errors/ \| \| 2 \| (diagnos* adj3 (accurac* or concordan* or agreement* or error* or discrepanc* or discord* or misclassif*)).tw. \| \| 3 \| (misdiagnos* or misclassif* or incorrect diagnos* or missed diagnos*).tw. \| \| 4 \| (referral* adj3 (diagnos* or accurac* or concordan* or agreement* or quality or appropriate* or inappropriate* or triage)).tw. \| \| 5 \| (triage adj3 (accurac* or appropriate* or quality or referral*)).tw. \| \| 6 \| 1 or 2 or 3 or 4 or 5 \| \| 7 \| exp Eye Diseases/ \| \| 8 \| exp Ophthalmology/ \| \| 9 \| (ophthalm* or ocular or eye disease* or eye disorder* or eye condition* or eye complaint* or visual symptom* or vision loss or red eye).tw. \| \| 10 \| 7 or 8 or 9 \| \| 11 \| exp Physicians/ \| \| 12 \| exp Physician Assistants/ \| \| 13 \| exp Health Personnel/ \| \| 14 \| exp Optometrists/ \| \| 15 \| exp Nurses/ \| \| 16 \| exp Primary Health Care/ \| \| 17 \| (non-ophthalmologist* or nonophthalmologist* or non-specialist* or nonspecialist*).tw. \| \| 18 \| (general practitioner* or GP or GPs or family physician* or primary care physician* or primary care provider* or primary care).tw. \| \| 19 \| (emergency physician* or emergency doctor* or emergency clinician* or emergency medicine physician*).tw. \| \| 20 \| (optometrist* or optician* or community optometry or community optometrist*).tw. \| \| 21 \| (nurse* or triage nurse* or physician associate* or physician assistant*).tw. \| \| 22 \| 11 or 12 or 13 or 14 or 15 or 16 or 17 or 18 or 19 or 20 or 21 \| \| 23 \| exp Emergency Service, Hospital/ \| \| 24 \| exp Emergency Room Visits/ \| \| 25 \| (emergency department* or emergency room* or accident and emergency or A&E or ED).tw. \| \| 26 \| (emergency eye adj3 (clinic* or department* or service*)).tw. \| \| 27 \| (eye casualty or ophthalmic casualty).tw. \| \| 28 \| (acute eye adj3 (clinic* or care or service*)).tw. \| \| 29 \| (urgent eye adj3 (clinic* or care or service*)).tw. \| \| 30 \| (ophthalmic emergency or ophthalmic emergencies or eye emergency or eye emergencies).tw. \| \| 31 \| (walk-in eye clinic* or walk in eye clinic*).tw. \| \| 32 \| (urgent ophthalmolog* or emergency ophthalmolog*).tw. \| \| 33 \| (red eye clinic* or minor injur* or urgent treatment centre* or same day emergency care).tw. \| \| 34 \| 23 or 24 or 25 or 26 or 27 or 28 or 29 or 30 or 31 or 32 or 33 \| \| 35 \| 6 and 10 and 22 and 34 \| |
| --- | --- | --- | --- | --- | --- | --- | --- | --- | --- | --- | --- | --- | --- | --- | --- | --- | --- | --- | --- | --- | --- | --- | --- | --- | --- | --- | --- | --- | --- | --- | --- | --- | --- | --- | --- | --- | --- | --- | --- | --- | --- | --- | --- | --- | --- | --- | --- | --- | --- | --- | --- | --- | --- | --- | --- | --- | --- | --- | --- | --- | --- | --- | --- | --- | --- | --- | --- | --- | --- | --- | --- | --- |

Two authors (L.G. and A.P.) will screen the titles and abstracts against the inclusion and exclusion criteria. The full text for studies that appear to comply with these criteria will then be screened. Any conflict will be discussed and resolved with the assistance of a third independent reviewer. Justification of exclusions will be recorded, and a PRISMA flow diagram drafted to depict search, screening, and inclusion results.
