# Supplementary material for: Accuracy of ophthalmic referral diagnoses by non-ophthalmologists in acute eye care: protocol for a systematic review and meta-analysis
Source: BMJ Open. 2026 Jun 12;16(6):e121497. doi: 10.1136/bmjopen-2026-121497 (PMC13289109; doi:10.1136/bmjopen-2026-121497)
Supplement: Supplementary file 2 [file bmjopen-2026-121497.reviewer_comments.pdf]

PEER REVIEW HISTORY

BMJ Open publishes all reviews undertaken for accepted manuscripts. Reviewers are asked to complete a checklist review form and are provided with free text boxes to elaborate on their assessment. These free text comments are reproduced below.

ARTICLE DETAILS

Title (Provisional)

Accuracy of Ophthalmic Referral Diagnoses by Non-Ophthalmologists in Acute Eye Care: Protocol for a Systematic Review and Meta-Analysis

Authors

Gil, Luis Cunha; Powis, Anaya; Wilson, Helen; Thampy, Reshma; Kousha, Obaid; Dhawahir-Scala, Felipe

---

VERSION 1 - REVIEW

**Reviewer**	**1**
**Name**	**Chan, Chi-Ming**
**Affiliation**	**Fu Jen Catholic University**
**Date**	**01-May-2026**
**COI**	**None**

I reviewed the manuscript, “Accuracy of Ophthalmic Referral Diagnoses by Non-Ophthalmologists in Acute Eye Care: Protocol for a Systematic Review and Meta-Analysis.” The manuscript is a protocol for a planned systematic review and meta-analysis evaluating diagnostic accuracy of ophthalmic referral diagnoses by non-ophthalmologists in acute eye care settings, with planned searches of MEDLINE, Embase, and CENTRAL, PROSPERO registration CRD420261352717, independent screening/extraction, QUADAS-2 risk-of-bias assessment, and planned synthesis of 2×2 diagnostic accuracy data.

The manuscript addresses an important and clinically meaningful question for acute ophthalmology, emergency medicine, and healthcare pathway design. The authors correctly identify that ophthalmic presentations account for approximately 1.46–6% of accident and emergency attendances, that prior single-centre studies suggest referral misdiagnosis rates of around one-third, and that inaccurate initial diagnosis may delay appropriate treatment of vision-threatening disorders such as acute angle closure glaucoma, retinal detachment, and microbial keratitis. The protocol proposes a systematic review registered with PROSPERO, searches of MEDLINE, Embase, and CENTRAL from inception to April 2025, independent screening and data extraction by two reviewers, QUADAS-2 risk-of-bias assessment, planned extraction of 2×2 diagnostic accuracy data, and subgroup analyses by referring clinician type

and anatomical location of pathology. The topic is highly suitable for BMJ Open, and the protocol is broadly promising. However, the present version still requires minor revision because several important methodological, diagnostic-accuracy, search-strategy, terminology, and reporting details remain insufficiently specified.

First, the protocol requires sharper definition of the review question and diagnostic target. The manuscript repeatedly refers to “diagnostic accuracy,” but it is not sufficiently clear whether the authors intend to evaluate exact diagnostic concordance, clinically acceptable diagnostic category agreement, identification of sight-threatening disease, appropriateness of referral urgency, or all of these as separate outcomes. These are not interchangeable. A referral diagnosis of “red eye” may be nonspecific but clinically appropriate if the patient is triaged urgently; conversely, a precise but wrong diagnosis may still direct the patient to the correct acute service. The authors should define the primary outcome more rigorously and clarify how they will treat partially correct, nonspecific, syndromic, anatomical, or urgency-based referral diagnoses. Without this clarification, the planned 2×2 framework may oversimplify the real clinical complexity of acute ophthalmic referrals.

Second, the planned diagnostic-accuracy synthesis is currently methodologically underdeveloped. The authors state that they will construct 2×2 tables with true positives, false positives, true negatives, and false negatives comparing referral diagnosis with final ophthalmologist diagnosis. However, in multi-disease referral settings, the definition of a “true negative” is not obvious. Diagnostic accuracy meta-analysis is usually most straightforward when there is one index test and one target condition, but this review appears to include a broad range of ophthalmic conditions across cornea, retina, anterior segment, conjunctiva, neuro-ophthalmology, orbit, and external eye. The authors should clarify whether they will analyse each disease category separately, collapse diagnoses into clinically meaningful groups, or calculate simple concordance proportions rather than sensitivity and specificity when true negatives cannot be validly defined. They should also consider whether hierarchical summary ROC or bivariate random-effects models would be more appropriate than a generic random-effects model if sensitivity and specificity are pooled.

Third, the search strategy needs refinement. The selected databases are reasonable, but the search terms shown in the supplementary strategy appear too narrow and may miss relevant studies. For example, terms such as “triage,” “referral quality,” “referral appropriateness,” “diagnostic concordance,” “misclassification,” “eye emergency,” “ophthalmic emergency,” “eye casualty,” “walk-in eye clinic,” “urgent ophthalmology,” “primary care,” and “community optometry” should be considered more systematically. The line containing “(((eye casualty or emergency eye department or red eye clinic or acute eye care or eye hospital or emergency department or accident) and emergency)…” also appears syntactically vulnerable and may not retrieve all intended records. The authors should work with an information specialist or librarian to ensure that the final strategy is reproducible, sensitive, and correctly adapted across MEDLINE, Embase, and CENTRAL. Restricting to

English-language full texts should also be justified, as this may introduce language bias in a review intended to examine referral accuracy across healthcare systems.

Fourth, eligibility criteria and population definitions need greater precision. The protocol includes “non-ophthalmologist healthcare professionals,” including emergency physicians, general practitioners, optometrists, nurses, physician associates, and other non-specialists. However, optometrists may have substantially different ophthalmic training from general practitioners or emergency physicians, and in some countries optometrists function as primary eye-care specialists. The authors should predefine whether optometrists will be analysed separately from other non-ophthalmologists. Similarly, emergency eye departments, eye casualty services, hospital emergency departments, community optometry pathways, and tele-triage services may have markedly different referral thresholds and diagnostic case mix. These settings should not be treated as equivalent without planned subgroup or narrative stratification. The authors should also clarify whether studies involving mixed referrer groups will be included only if data for non-ophthalmologists can be separated.

Fifth, risk-of-bias and certainty assessment require more detail. QUADAS-2 is an appropriate tool, but it should be adapted explicitly to this review. The authors should specify what will constitute low, high, or unclear risk for patient selection, index diagnosis, reference standard, and flow/timing in the context of referral-diagnosis studies. For example, it matters whether the ophthalmologist making the final diagnosis was blinded to the referral diagnosis, whether the final diagnosis was made immediately or after investigations/follow-up, whether all referred patients were included consecutively, and whether uncertain or missing diagnoses were excluded. The proposed use of GRADE is reasonable, but for diagnostic accuracy reviews the authors should specify that certainty will be assessed for each main diagnostic outcome or diagnostic category, rather than as a single global rating for the entire review. Publication bias assessment with Egger test and funnel plots should be used cautiously, as these methods are not always appropriate for diagnostic accuracy meta-analysis and are often unreliable with heterogeneous accuracy measures.

Sixth, the manuscript would benefit from broader contextualization within acute ophthalmology, vision-threatening disease pathways, and evidence-synthesis methodology. The following studies may be helpful for consideration because they relate to ophthalmic emergencies, retinal and corneal conditions, diagnostic or therapeutic decision-making, and systematic review/meta-analysis methodology in ophthalmology: 1. PMID: 41616875; doi: 10.1016/j.ijantimicag.2025.107707. This study may be relevant because fungal keratitis represents a potentially vision-threatening corneal condition in which diagnostic delay or incorrect referral can have serious consequences. 2. PMID: 41991784; doi: 10.1007/s15010-026-02772-4. This study may be relevant because infectious endophthalmitis is an acute, high-stakes ophthalmic condition where early recognition and urgent referral are clinically important. 3.PMID: 40419166; doi: 10.1016/j.survophthal.2025.05.008. This study may be relevant because retinal vascular occlusive disease is an important acute retinal presentation

and illustrates the role of evidence synthesis in guiding ophthalmic treatment pathways. 4. PMID: 41689131; doi: 10.1186/s40001-026-03868-1. This study may be relevant because glaucoma-related presentations and surgical decision-making are clinically important in ophthalmology, and acute glaucoma is specifically mentioned as a vision-threatening referral diagnosis in the protocol.

The manuscript also requires careful language and formatting revision. There are several typographical and grammatical problems, including “general practioners,” “University of St Andrews, North Haugh... Department of Ophthalmology, Ninewells Hospital & Medical School is Ninewells Avenue,” “although accounting only around 5%,” “from onset,” and inconsistent spacing around “PRISMA -P” and “2x2.” The protocol should also avoid overclaiming that this will definitively be the first review unless the authors have confirmed this through a scoping search. In addition, the “Types of interventions” section is awkward for a diagnostic accuracy review; it would be clearer to refer to the “index diagnosis” or “index referral diagnosis” rather than an intervention.

Overall, the manuscript has merit and the core question may be of interest to readers, but the present version still contains notable deficiencies in diagnostic-outcome definition, methodological description, search-strategy construction, diagnostic-accuracy synthesis planning, risk-of-bias interpretation, and language quality. I therefore recommend minor revision. The work could become suitable for publication after careful correction of the points above.

VERSION 1 - AUTHOR RESPONSE

Reviewer 1 Comments

Reviewer: Dr. Chi-Ming Chan, Fu Jen Catholic University

Comment 1

Comment:

> First, the protocol requires sharper definition of the review question and diagnostic target. The manuscript repeatedly refers to “diagnostic accuracy,” but it is not sufficiently clear whether the authors intend to evaluate exact diagnostic concordance, clinically acceptable diagnostic category agreement, identification of sight-threatening disease, appropriateness of referral urgency, or all of these as separate outcomes. These are not interchangeable. A referral diagnosis of “red eye” may be nonspecific but clinically appropriate if the patient is triaged urgently; conversely, a precise but wrong diagnosis may still direct the patient to the correct acute service. The authors should define the primary outcome more rigorously and clarify how they will treat partially correct, nonspecific, syndromic, anatomical, or urgency-based referral diagnoses. Without this clarification, the planned 2×2 framework may oversimplify the real clinical complexity of acute ophthalmic referrals.

Response:

We appreciate the reviewer’s observation that our description of diagnostic accuracy and primary outcome require stricter definitions to avoid oversimplification in our analysis. In response, we have revised the ‘Index diagnosis’, ‘Comparison’ and ‘Types of Outcomes’ sections to reflex our updated outcome measures. Our primary outcome is now defined as diagnostic concordance between the

referral diagnosis made by a non-ophthalmologist and the final diagnosis confirmed by an ophthalmologist. We acknowledge that not all studies will record results in binary categories of concordant and non-concordant, so we have clarified that outliers to these will have definitions extracted as per the study.

Comment 2

Comment:

> Second, the planned diagnostic-accuracy synthesis is currently methodologically underdeveloped. The authors state that they will construct 2×2 tables with true positives, false positives, true negatives, and false negatives comparing referral diagnosis with final ophthalmologist diagnosis. However, in multi-disease referral settings, the definition of a "true negative" is not obvious. Diagnostic accuracy meta-analysis is usually most straightforward when there is one index test and one target condition, but this review appears to include a broad range of ophthalmic conditions across cornea, retina, anterior segment, conjunctiva, neuro-ophthalmology, orbit, and external eye. The authors should clarify whether they will analyse each disease category separately, collapse diagnoses into clinically meaningful groups, or calculate simple concordance proportions rather than sensitivity and specificity when true negatives cannot be validly defined. They should also consider whether hierarchical summary ROC or bivariate random-effects models would be more appropriate than a generic random-effects model if sensitivity and specificity are pooled.

Response:

We thank the reviewer for highlighting important concerns regarding the planned diagnostic accuracy synthesis in a heterogeneous, multi-condition referral setting. In response, we have revised the Data Synthesis, Types of Outcomes, Meta-Bias, and abstract sections to clarify the analytic hierarchy and improve methodological transparency. We now explicitly acknowledge that true negatives cannot be validly defined across the full multi-condition referral case mix, and have therefore designated pooled diagnostic concordance proportions as the primary quantitative outcome across all included studies. Construction of 2×2 contingency tables and calculation of condition-specific sensitivity and specificity are now clearly framed as a secondary analysis, undertaken only where studies report data for a clearly defined target condition permitting valid diagnostic classification. Bivariate random-effects or hierarchical summary ROC methods will be considered where sufficient condition-specific sensitivity and specificity data are available. The Meta-Bias section has also been revised to reframe funnel plots and the Egger test as supplementary exploratory analyses given their well-recognised limitations in diagnostic accuracy and concordance data, with qualitative comparison of reported outcomes against study methods designated as the primary approach to assessing publication bias. The abstract Methods and Analysis section has also been revised to reflect these methodological changes and to ensure consistency with the revised concordance-focussed analysis.

Comment 3

Comment:

> Third, the search strategy needs refinement. The selected databases are reasonable, but the search terms shown in the supplementary strategy appear too narrow and may miss relevant studies. For example, terms such as "triage," "referral quality," "referral appropriateness," "diagnostic concordance," "misclassification," "eye emergency," "ophthalmic emergency," "eye casualty," "walk-in eye clinic," "urgent ophthalmology," "primary care," and "community optometry" should be considered more systematically. The line containing "(((eye casualty or emergency eye department or red eye clinic or acute eye care or eye hospital or emergency department or accident) and emergency)…" also appears syntactically vulnerable and may not retrieve all intended records. The authors should work with an information specialist or librarian to ensure that the final strategy is reproducible, sensitive, and correctly adapted across MEDLINE, Embase, and CENTRAL.Restricting to English-language full texts should also be justified, as this may introduce language bias in a review intended to examine referral accuracy across healthcare systems.

Response:

We thank the reviewer for highlighting limitations in the original search strategy. We have revised the proposed MEDLINE strategy to include additional terms for triage, referral quality, referral appropriateness, diagnostic concordance, misclassification, primary care, community optometry, eye casualty, walk-in eye clinics, urgent ophthalmology and ophthalmic emergency care. The previously syntactically vulnerable acute-care line has been rectified. The revised strategy will be adapted for Embase and CENTRAL and checked against known relevant studies. A justification for the English language only restriction has now been included in the methods under 'Types of Studies' subheading, and the implications of this have been acknowledged as a point under the Strengths & Limitations section of the paper.

Comment 4

Comment:

> Fourth, eligibility criteria and population definitions need greater precision. The protocol includes "non-ophthalmologist healthcare professionals," including emergency physicians, general practitioners, optometrists, nurses, physician associates, and other non-specialists. However, optometrists may have substantially different ophthalmic training from general practitioners or emergency physicians, and in some countries optometrists function as primary eye-care specialists. The authors should predefine whether optometrists will be analysed separately from other non-ophthalmologists. Similarly, emergency eye departments, eye casualty services, hospital emergency departments, community optometry pathways, and tele-triage services may have markedly different referral thresholds and diagnostic case mix. These settings should not be treated as equivalent without planned subgroup or narrative stratification. The authors should also clarify whether studies involving mixed referrer groups will be included only if data for non-ophthalmologists can be separated.

Response:

We agree with the reviewer comments and thank them for identifying that the eligibility criteria and population definition needed clarification. The manuscript has been updated to reflect the variability in the role of optometrists across countries and thus that their data extraction and analysis will be done in an independent subgroup to the other collated non-ophthalmologists. We also acknowledge the differences in referral threshold depending on clinical setting and have therefore delineated between them to enable subgroup analysis. The treatment of studies involving mixed referrer groups has been clarified in the methods also.

Comment 5

Comment:

> Fifth, risk-of-bias and certainty assessment require more detail. QUADAS-2 is an appropriate tool, but it should be adapted explicitly to this review. The authors should specify what will constitute low, high, or unclear risk for patient selection, index diagnosis, reference standard, and flow/timing in the context of referral-diagnosis studies. For example, it matters whether the ophthalmologist making the final diagnosis was blinded to the referral diagnosis, whether the final diagnosis was made immediately or after investigations/follow-up, whether all referred patients were included consecutively, and whether uncertain or missing diagnoses were excluded. The proposed use of GRADE is reasonable, but for diagnostic accuracy reviews the authors should specify that certainty will be assessed for each main diagnostic outcome or diagnostic category, rather than as a single global rating for the entire review. Publication bias assessment with Egger test and funnel plots should be used cautiously, as these methods are not always appropriate for diagnostic accuracy meta-analysis and are often unreliable with heterogeneous accuracy measures.

Response:

We thank the reviewer for highlighting that QUADAS-2 should be explicitly adapted to the context of referral-diagnosis studies. We agree that simply stating use of QUADAS-2 was insufficient. We have therefore revised the Risk of Bias section to specify how each QUADAS-2 domain will be interpreted in this review: patient selection, index diagnosis, reference standard, and flow/timing. In particular, we now clarify how risk of bias will be judged in relation to representative referral sampling, documentation of

the referral diagnosis before specialist assessment, reliability of the ophthalmologist reference diagnosis, timing between referral and specialist assessment, missing diagnoses, and exclusions from analysis. We agree that certainty of evidence should not be presented as a single global rating for the review. We have revised the manuscript to state that GRADE will be applied separately to each main diagnostic outcome or diagnostic category, reflecting its outcome-specific application. We have also revised the Meta-Bias section to avoid over-reliance on Egger tests and funnel plots.

Comment 6

Comment:

Sixth, the manuscript would benefit from broader contextualization within acute ophthalmology, vision-threatening disease pathways, and evidence-synthesis methodology. The following studies may be helpful for consideration because they relate to ophthalmic emergencies, retinal and corneal conditions, diagnostic or therapeutic decision-making, and systematic review/meta-analysis methodology in ophthalmology: 1. PMID: 41616875; doi: 10.1016/j.ijantimicag.2025.107707. This study may be relevant because fungal keratitis represents a potentially vision-threatening corneal condition in which diagnostic delay or incorrect referral can have serious consequences. 2. PMID: 41991784; doi: 10.1007/s15010-026-02772-4. This study may be relevant because infectious endophthalmitis is an acute, high-stakes ophthalmic condition where early recognition and urgent referral are clinically important. 3.PMID: 40419166; doi: 10.1016/j.survophthal.2025.05.008. This study may be relevant because retinal vascular occlusive disease is an important acute retinal presentation and illustrates the role of evidence synthesis in guiding ophthalmic treatment pathways. 4. PMID: 41689131; doi: 10.1186/s40001-026-03868-1. This study may be relevant because glaucoma-related presentations and surgical decision-making are clinically important in ophthalmology, and acute glaucoma is specifically mentioned as a vision-threatening referral diagnosis in the protocol.

Response:

We thank the reviewer for this helpful suggestion. We have revised the Introduction to provide broader contextualisation within acute ophthalmology, emergency referral pathways, and the clinical consequences of delayed or inaccurate diagnosis. Specifically, we expanded discussion of increasing demand on ophthalmic services and the growing role of community-based healthcare professionals in acute eye care pathways. We also strengthened the clinical rationale for accurate referral diagnosis by broadening the discussion of vision-threatening and systemically significant ophthalmic emergencies, now including endophthalmitis and retinal vascular occlusive disease alongside acute angle-closure glaucoma, retinal detachment, and microbial keratitis. In addition, we emphasised that ocular findings may provide important diagnostic information relevant to other specialties and clarified the impact of referral diagnostic accuracy on triage urgency, timeliness of specialist assessment, and initiation of management. Finally, we revised the final paragraph of the Introduction to more clearly define the current evidence gap and justify the need for a systematic review and meta-analysis in this area.

Comment 7

Comment:

The manuscript also requires careful language and formatting revision. There are several typographical and grammatical problems, including “general practioners,” “University of St Andrews, North Haugh... Department of Ophthalmology, Ninewells Hospital & Medical School is Ninewells Avenue,” “although accounting only around 5%,” “from onset,” and inconsistent spacing around “PRISMA -P” and “2x2.” The protocol should also avoid overclaiming that this will definitively be the first review unless the authors have confirmed this through a scoping search. In addition, the “Types of interventions” section is awkward for a diagnostic accuracy review; it would be clearer to refer to the “index diagnosis” or “index referral diagnosis” rather than an intervention.

Response:

We thank the reviewer for helpfully identifying the language and grammatical errors in the original manuscript. These have now been rectified as per the reviewer’s recommendation. The claim that this is the first definitive review of its kind has been tempered as suggested. We agree with the reviewer

that the term “intervention” is inappropriate for this review, which evaluates diagnostic concordance rather than treatment effects. We have revised the eligibility criteria terminology from “Types of interventions” to “Index diagnosis”, and from “Types of control” to “Reference standard”, in keeping with diagnostic accuracy review terminology. The Methods section now clarifies that the index diagnosis is the initial referral diagnosis made by a non-ophthalmologist, while the reference standard is the final diagnosis established by an ophthalmologist.

VERSION 2 - REVIEW

**Reviewer**	**1**
**Name**	**Chan, Chi-Ming**
**Affiliation**	**Fu Jen Catholic University**
**Date**	**28-May-2026**
**COI**	

Good work to be accepted.
